# Principles of good practice for concept definition in the context of translation and linguistic validation of clinical outcome assessments (COAs)

**DOI:** 10.1186/s41687-025-00920-2

**Published:** 2025-07-14

**Authors:** Benjamin Arnold, Dana Weiss, Emily Parks-Vernizzi, Barbara Brandt, Ana Popielnicki, Beatrice Tedeschi, Clayon Hamilton, Mark Wade, John Chaplin, Holger Muehlan, Jussi P. Repo, Sonya Eremenco

**Affiliations:** 1 FACITtrans, 151 Bay Cove Drive, Ponte Vedra, FL 32082 USA; 2 Formerly of Mapi Research Trust, Lyon, France; 3 Critical Path Institute, Tucson, AZ USA; 4 GSK, Collegeville, PA USA; 5 Lionbridge, Didcot, England; 6 Faculty of Health Sciences, Simon Fraser University, British Columbia, Burnaby, Canada; 7 TransPerfect New York, New York, USA; 8 Sahlgrenska Academy at Gothenburg University, Gothenburg, Sweden; 9 Health & Medical University, Erfurt, Germany; 10 Tampere University Hospital and University of Tampere, Tampere, Finland

**Keywords:** Concept definition, Item definition, Concept elaboration, Linguistic validation, Translation, Cultural adaptation, Clinical outcome assessment, Patient-reported outcome, Best practice

## Abstract

**Background:**

Translation teams conducting translation and cultural adaptation find it paramount to properly describe concepts of items within clinical outcome assessments (COAs). To minimize potential threats to linguistic/conceptual equivalence, these teams must understand the concepts a COA intends to measure. This research provides recommendations for the process of developing concept definitions in general, as well as specific recommendations on who should be involved in the process and what a concept definition document should contain.

**Methods:**

The Concept Definition Working Group of the International Society for Quality of Life Research (ISOQOL) Translation and Cultural Adaptation Special Interest Group (TCA-SIG) carried out a literature review and a survey of 20 professionals working in the area of translation and linguistic validation of COAs. The Working Group based recommendations on a combination of survey results and consensus building via online meetings.

**Results:**

Translation teams should develop concept definitions during the preparation phase of the translation process, assuming they do not already exist, and include COA developers and project managers with experience in linguistic validation and conceptual analysis of COAs. The Working Group recommends that concept definitions consist of information related to the therapeutic area being studied, information related to the COA development process, definitions of concepts and domains, as well as elaboration of colloquialisms and acceptable/unacceptable translation alternatives. We recommend centralized distribution of concept definitions.

**Conclusions:**

Concept definitions guide stakeholders and ensure all parties align on the intended meaning of items being translated. While experts have made recommendations for best practices around translation and linguistic validation methodology, they have not clearly delineated the process of defining concepts. The Concept Definition Working Group of the ISOQOL TCA-SIG has therefore developed a set of recommendations for the process of defining concepts. With these recommendations the Working Group intends to standardize the development of concept definitions with the goal of enhancing conceptual equivalence across translations to support data pooling and provide confidence that clinical trial data are comparable, interpretable, and can be relied upon in evaluating clinical benefit of treatments.

**Supplementary Information:**

The online version contains supplementary material available at 10.1186/s41687-025-00920-2.

## Background

A continued interest in incorporating the voice of the patient into multinational clinical trials and research studies has led to the need for translated versions of not only patient-reported outcome measures (PROMs), but also clinical outcome assessments (COAs) as a whole. In light of the increase in patient-centered initiatives addressing the impact of medical treatments on a global scale, the need for obtaining the perspectives of patients from diverse linguistic and cultural backgrounds has become critical [1]. The U.S. Food and Drug Administration (FDA) has identified four types of COAs which are intended to describe the way a patient functions, feels, and survives: patient-reported outcome (PRO), observer-reported outcome (ObsRO), clinician-reported outcome (ClinRO), and performance outcome (PerfO) measures [2]. Regulatory bodies, such as the FDA and the European Medicines Agency (EMA), have shown a growing interest in evidence of the linguistic/conceptual equivalence produced by the translation and linguistic validation process [3]. Acquadro and colleagues define a meaningful translation as one that is “conceptually equivalent to the source text and culturally and linguistically appropriate in the target country to facilitate the pooling and comparison of data” [4]. Differences measured should indicate true differences between groups being evaluated as opposed to bias introduced by the tools being used to measure them [5].

Although achieving 100% equivalence across languages and cultures is impossible, understanding and identifying the concepts that a COA intends to measure is of the utmost importance to minimize potential threats to equivalence. The importance of properly describing concepts is paramount for translation teams conducting translation and cultural adaptation of COAs. Considering that establishing the meaning of the source language is frequently problematic when COAs are translated, the threat imposed on data validity is magnified [6].

While experts have recommended best practices around translation and linguistic validation methodology for PROMs, with the most impactful being the ISPOR Task Force for Translation and Cultural Adaptation’s ‘Principles of Good Practice for the Translation and Cultural Adaptation Process for Patient-Reported Outcome (PRO) Measures’ [7], the literature has not clearly delineated the process of defining concepts, i.e., what each COA item is intended to measure. A number of publications discuss its importance to varying degrees. The ISPOR Task Force’s Principles of Good Practice refer to a “Preparation Phase” including definitions for concepts used in the measure. Similarly, Acquadro and colleagues point to a “Preparation Phase” including the itemization of source text to clarify the meaning of the source language to promote a higher probability that translation teams will understand the concepts as intended by the developer [4]. Clifton et al. discuss the need for construct and measure-specific advice to be transferred from developers to translators in a formal, detailed, and accessible way [8]. Hawkins et al. recommend detailed descriptions of item intents, which can support translation teams in maximizing equivalence while maintaining the linguistic and cultural veracity of the target language [9], while the Critical Path Institute’s PRO Consortium mentions generating an “Item Definition Table” describing the concepts being measured as part of the COA development process [1] to document this information during the qualitative phase. In a survey carried out by McKown et al., 93% of respondents representing a cross-section of experts in the area of cross-cultural COA linguistic validation endorsed the need for a concept definition document [10].

While it is clear that defining concepts in the context of COA translation and linguistic validation is an invaluable tool, recommendations around the concept definition process do not exist. The Concept Definition Working Group of the International Society for Quality of Life Research (ISOQOL) Translation and Cultural Adaptation Special Interest Group (TCA-SIG) has therefore developed these recommendations to address this gap in the literature. Without standardized recommendations for development of concept definitions, the process can vary widely from one Language Service Provider (LSP) to another, leading to suboptimal results.

## Methods

**Fig. 1:**
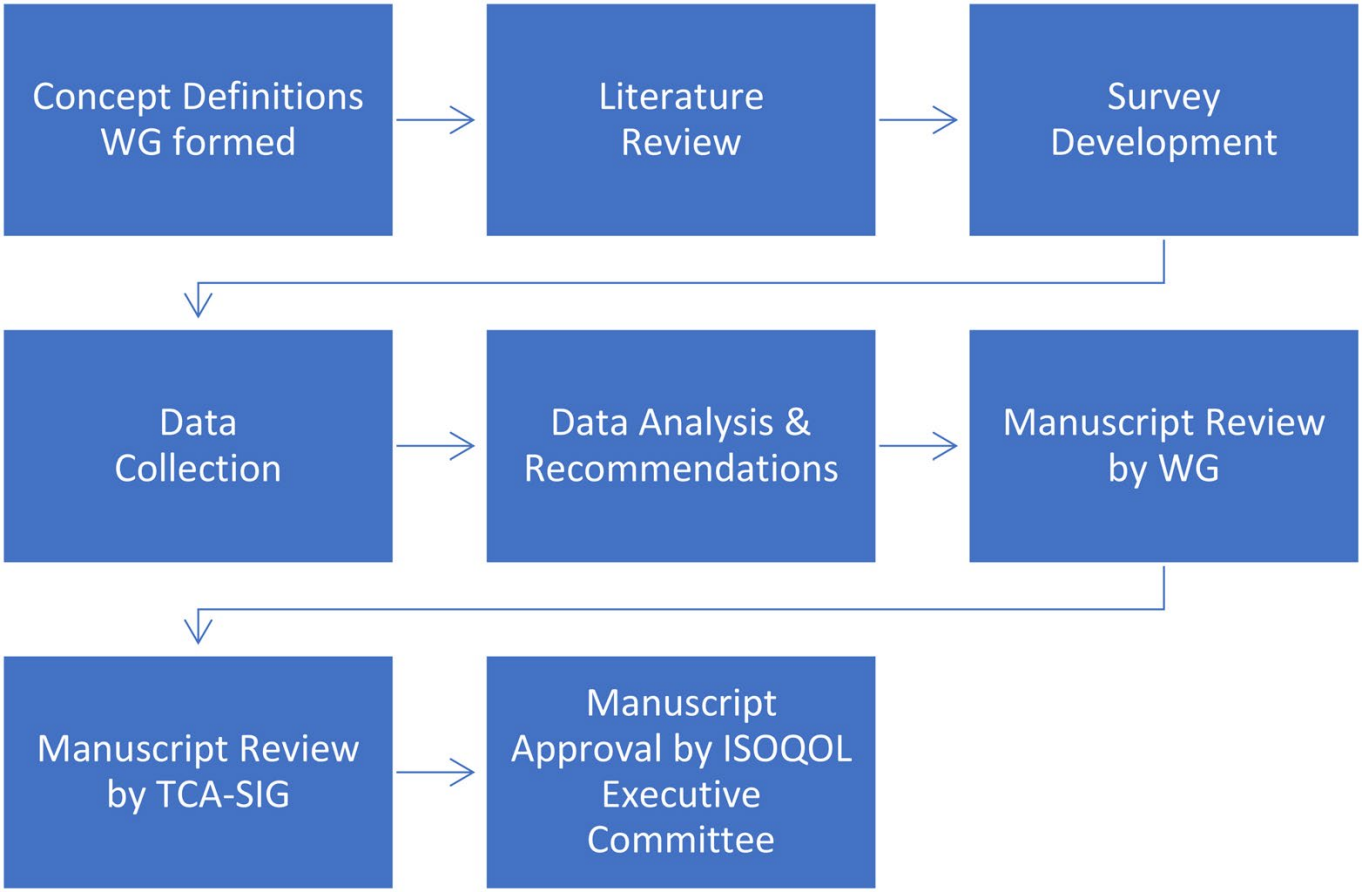
Process workflow for development of recommendations

Prompted by a survey request to develop good practice recommendations on concept definitions for COAs, the TCA-SIG formed a Working Group during the ISOQOL 2017 Annual Conference in Philadelphia, Pennsylvania, U.S. Members of the TCA-SIG with experience in concept definition development formed the Working Group, providing a good combination of backgrounds including LSPs, non-profit organizations, academia, COA developers, and the pharmaceutical industry. The members performed all work on a voluntary basis and based authorship on guidelines set forth by the International Committee of Medical Journal Editors (ICMJE) [11]. Figure 1 shows the steps included in the process.

The Working Group conducted a literature review to identify any existing recommendations or guidelines around the process of developing concept definitions. The Working Group searched the ISPOR Scientific Database, PubMed, and Google Scholar spanning from 1946 to present day using keywords identified by the Working Group (COA, concept definitions, concept elaborations, item definitions, linguistic validation). In addition, the team conducted a survey [Appendix A] to collect information from professionals who were identified by the Working Group as representing a diverse range of expertise based on experience with questionnaire development and/or translation and linguistic validation. The survey aimed to gather information regarding current practices and identify potential recommendations for the Working Group to consider. The Working Group developed and tested the survey content over the course of several online meetings.

The Working Group carried out the survey online via Survey Monkey, in English, and included 15 questions on topics specific to concept definition development and usage, such as frequency, process, content, target language, and involvement of the COA developer. The survey included four questions with a 5-point frequency-based verbal rating scale (“Always,” “Most of the time,” “Sometimes,” “Seldom,” “Never”). Four questions had dichotomous (i.e., “Yes”, “No”) response options, and the remaining seven questions had response options which were specific to the question being asked, some of which had a free-text option in which the respondent could elaborate on their answers. The last item invited additional comments to cover anything that was not yet covered by the survey questions. The Working Group sent a survey link to representatives of LSPs, eCOA providers, pharmaceutical companies and instrument developer/copyright holders. All recipients were English-speaking.

The Working Group analyzed the data descriptively. This provided the ability to discuss and base recommendations not only on their expertise, but also on areas in which there was agreement/disagreement among respondents. The Working Group considered the survey responses and developed recommendations based on further discussion and consensus building during two online meetings. The Working Group achieved consensus on recommendations without significant disagreement through discussion of differing perspectives until a shared agreement was reached.

The Working Group reviewed the first draft of the manuscript. After discussing and implementing feedback, the TCA-SIG reviewed the next draft, followed by additional revisions to address new feedback. The ISOQOL Executive Committee reviewed and approved the manuscript as the last step.

## Results

A total of 20 professionals with experience in the area of translation and linguistic validation of COAs provided responses to the TCA-SIG Concept Definition Survey between March and November 2020. A total of 18 respondents voluntarily provided organizational data (cApStAn LQA & LQC LA, Clayson Linguistic Validation Services Ltd, Critical Path Institute, EORTC, FACITtrans, GRC-Health.com, ICON Language Services, Oxford University Innovation, RWS Life Sciences, Santium, Swinburne University of Technology, TransPerfect, VeLo Language Services, Vitaccess Ltd, and Welocalize). One respondent self-identified as being a “lead in multiple pharma companies.” All 20 respondents answered questions 1–9. A total of 17 respondents answered question 14, and 19 respondents answered questions 10, 11, 12, 13, and 15. Respondents were not required to answer all questions, and the Working Group did not collect reasons for skipping items. See Supplemental Material for response frequencies by question and Table 1 for a concept definition example.

**Table 1:** Example concept definition

Instrument	Item code	Source	Concept	Term definitions	Acceptable/unacceptable translation alternatives
FACT-V [13]	V7	I have trouble bending	The idea of this item is to ask if the patient experiences difficulty inclining his/her body into a curved position, as one would when doing ordinary activities such as housework or gardening. Specific to the term “bending,” the developer confirmed that this concept came directly from interviews with vulvar cancer patients in which women explained that they had trouble bending to do housework or gardening. This difficulty with bending can be caused by a variety of symptoms including soreness/tenderness/narrowness in the vulva, but also soreness in the groin, and/ or lymphoedema depending on the patients. Translations should allow for multiple meanings of “bending,” i.e., bending into any position (forward, backwards, either side) and with any part of the body (e.g., from the waist or knees). Moreover, a description of the concept “bend” would be an acceptable alternative.	N/A	Acceptable alternative for “have trouble”: “have difficulty” Acceptable alternative for “bend”: “moving my body into a curved or inclined position from my waist or from my knees” Acceptable alternative for this item, if necessary: “It is difficult for me to bend”

### Results for the process of defining concepts in general

Regarding what to call the document containing the concept definitions, a total of 16/20 respondents indicated they referred to such a document not as “Concept definitions” or “Concept definition documents,” but as something else. “Concept elaborations” or “Concept elaboration documents” was mentioned by 10 of these 16 respondents. Through discussion, the Working Group decided that the name “Concept definitions” was most appropriate and would be maintained for the purpose of this manuscript. The Working Group deemed the term “Concept definitions” to be more specific in establishing the essential meaning of the terms and ideas present in the source, allowing for more nuanced explanation of what is desirable/undesirable in the translations. While other terms may be used, we view this as a step towards standardization of terminology.

**Fig. 2:**
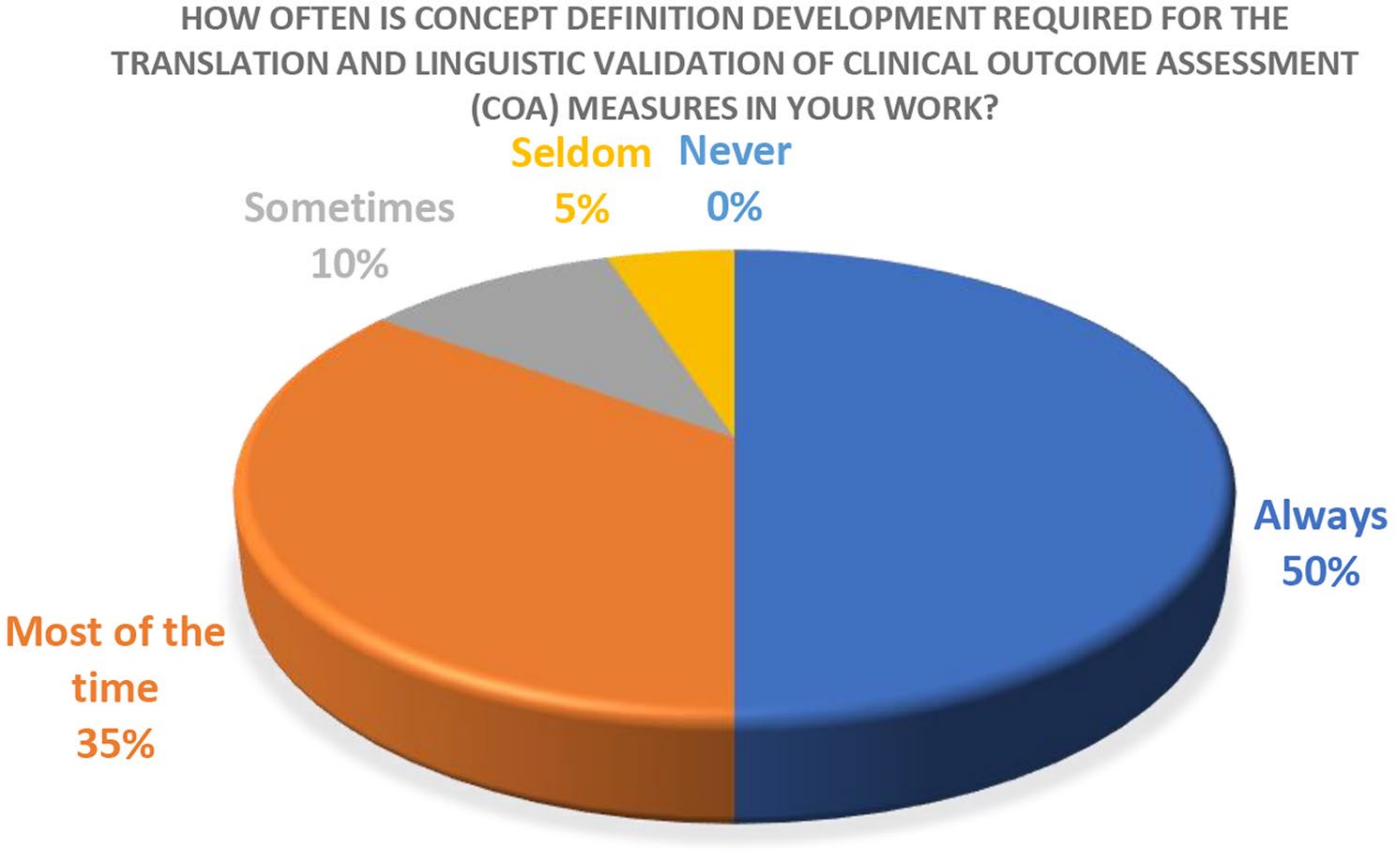
How often is concept definition required

Overall, 17/20 respondents agreed that concept definitions are required “Always” or “Most of the time” in the context of translation and linguistic validation of COAs. Two respondents reported “Sometimes” requiring them, and one respondent reported “Seldom” requiring them (Fig. 2).

Respondents reported English as the most common COA source language, with 15/20 stating it is “Seldom” or “Never” a language other than English. Most (18/20) respondents reported investigating whether such a document already existed for the COA requiring translation via consultation with the developer or copyright holder so as not to duplicate efforts. The majority (16/20) of respondents reported maintaining a repository of concept definitions.

Regarding the format used to produce concept definitions, 9/19 respondents indicated the use of Word tables and 7/19 indicated the use of Excel spreadsheets. Two respondents indicated using technology such as term bases and/or translation memory software.

**Fig. 3:**
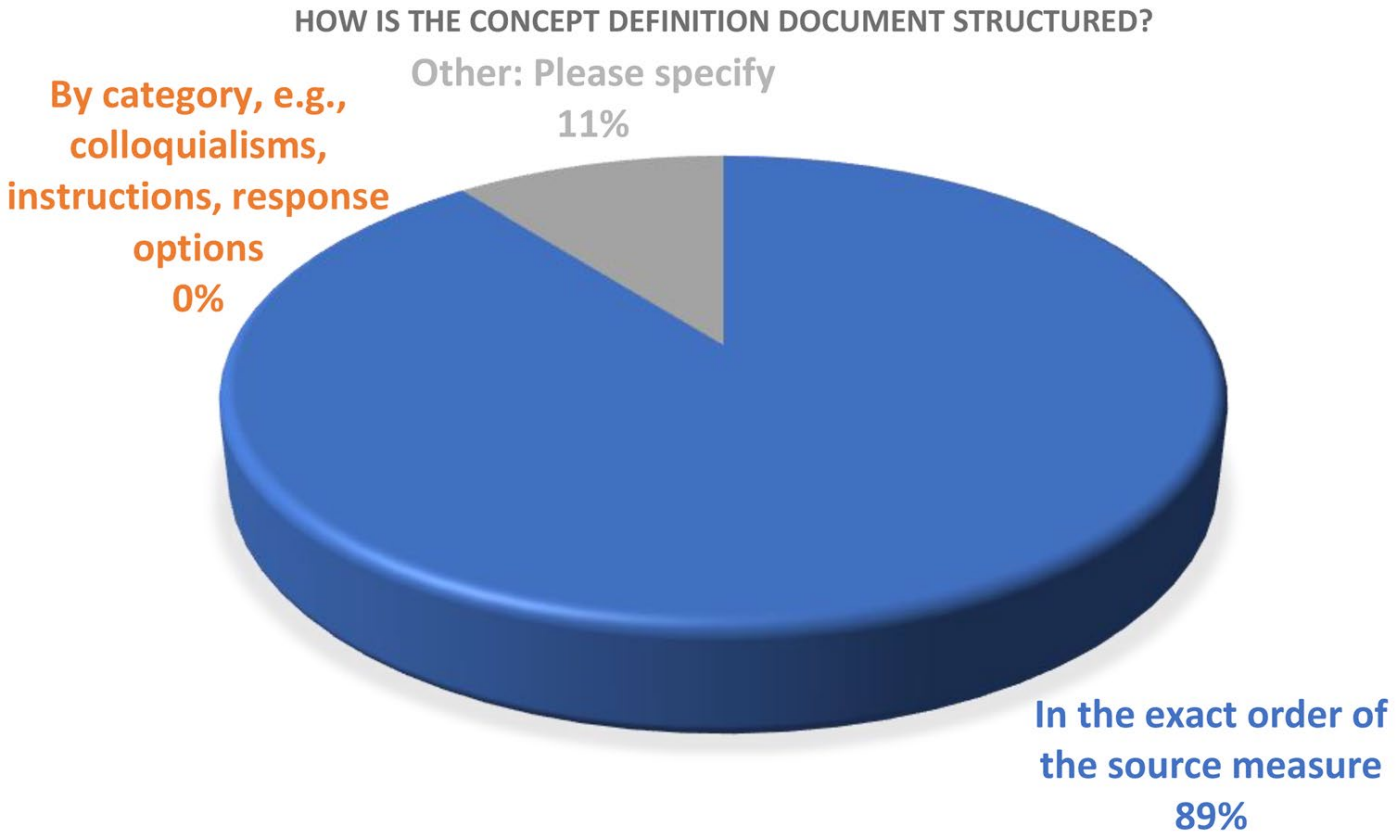
Concept definition structure

There was also agreement regarding the structure of the document, with 17/19 indicating that the concept definitions should follow the exact item sequence of the COA requiring translation. One respondent mentioned organizing by subscale and another respondent indicated that it depended on the way the concept definitions would be used (Fig. 3).

Most (17/19) respondents reported that updates to concept definition documents are made as needed throughout the life of a project. The other two respondents indicated that updates were made only after a project was completed.

### Recommendations for the process of defining concepts in general

Based on these findings, Table 2 lists the Working Group’s general recommendations for the process of defining concepts. Translation teams should create concept definitions for all COAs that may eventually be translated. If a COA has been translated previously, there is a good chance that concept definitions already exist, assuming the translation process was compliant with ISPOR good practice recommendations. In such cases, the Working Group recommends sourcing, reviewing, and adjusting these documents as necessary to convey the requisite information about the COA.

**Table 2:** General recommendations for the process of defining concepts:

Recommendation	Rationale
Involve the COA developer when possible. Involve a subject matter expert and/or a survey research expert when developer involvement not possible	Ensures information included in the concept definitions is as accurate and complete as possible
Check if concept definitions (or similar) exist	Enables leveraging of existing work and prevents duplication of efforts
Maintain a repository. Centralize distribution whenever possible	Protects intellectual property and encourages proper use of concept definitions (e.g., version control)
Adhere to item sequence of COA when feasible	Facilitates finding information within the concept definition document
Update concept definition document as soon as new information is available	Ensures everyone has the most up to date information
Seek client review/approval, when applicable	Ensures all relevant parties are informed and in agreement

The Working Group recommends centralized distribution of concept definitions. As these documents are derivative works, the same copyright recommendations made by Anfray and colleagues [12] for COAs should apply to concept definitions. We recommend, as good practice, that copyright holders/COA developers track and coordinate concept definitions in the same way they track and coordinate translations. Centralized distribution of concept definitions will not only help preserve the integrity of COA translations, but it will also encourage proper use of the correct version of the concept definitions, avoid the development of multiple versions of concept definitions for the same COA, and provide easy access to users, e.g., linguist/translators translating the COA. The Working Group recognizes that this recommendation can be difficult to fulfill for COAs in the public domain and in cases in which the developer does not have the resources for this level of coordination. Anfray et al. [12] provide strategies and additional information related to centralized distribution.

### Results for stakeholders involved in the process of defining concepts

Respondents provided varied responses regarding how many individuals are involved in creating the concept definition document. Overall, 8/20 respondents reported having two people involved in the process with 6/20 respondents reporting the involvement of three people. There was some support (4/20) for having four or more people involved, and minimal support (2/20) for including just one person in the concept definition development process. The majority (18/20) of respondents indicated that the concept definition writer is “Always” or “Most of the time” a native speaker of the source language of the COA requiring translation.

**Fig. 4:**
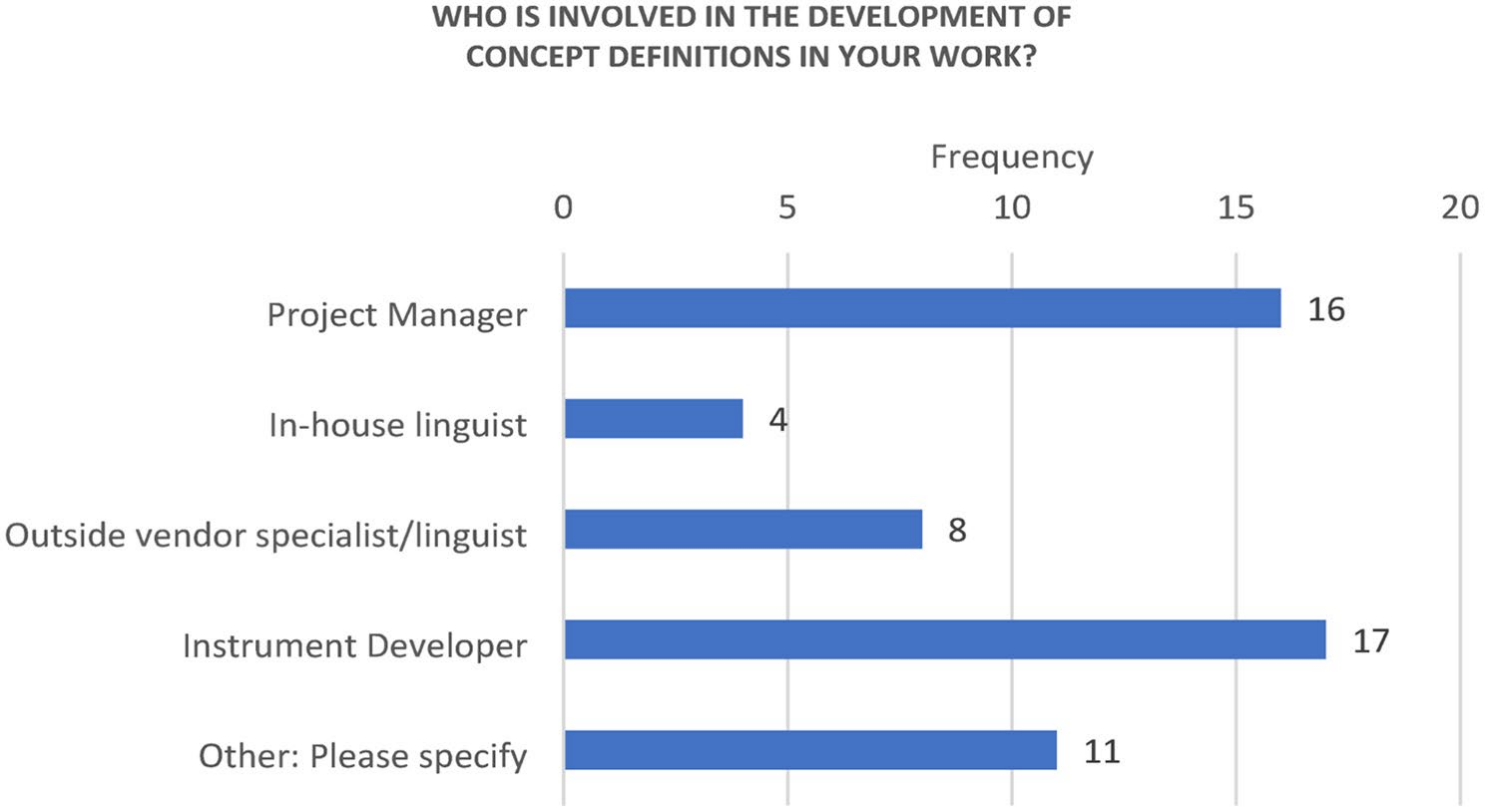
Stakeholders involved in concept definition development

In terms of the roles involved, 16/20 respondents indicated that a “Project manager” was involved, 4/20 respondents indicated an “in-house linguist” was involved, 8/20 respondents indicated including an “Outside vendor specialist/linguist,” and 17/20 respondents indicated including the “Instrument developer” in the process. In free text responses, one respondent each also mentioned including an “in-house COA specialist,” “app developers (if it is an eCOA measure),” “in-house survey research analyst,” “domain experts,” “patients, community members, healthcare professionals, sometimes policy makers,” “client,” “clinicians, patients,” “substantive editor (local clinical specialist – physician or psychologist) if measure is a ClinRO),” and “contract research partner conducting the instrument development and validation work.” Varying terminologies and structures across organizations could explain some of the variation seen in the responses to this question (Fig. 4).

**Fig. 5:**
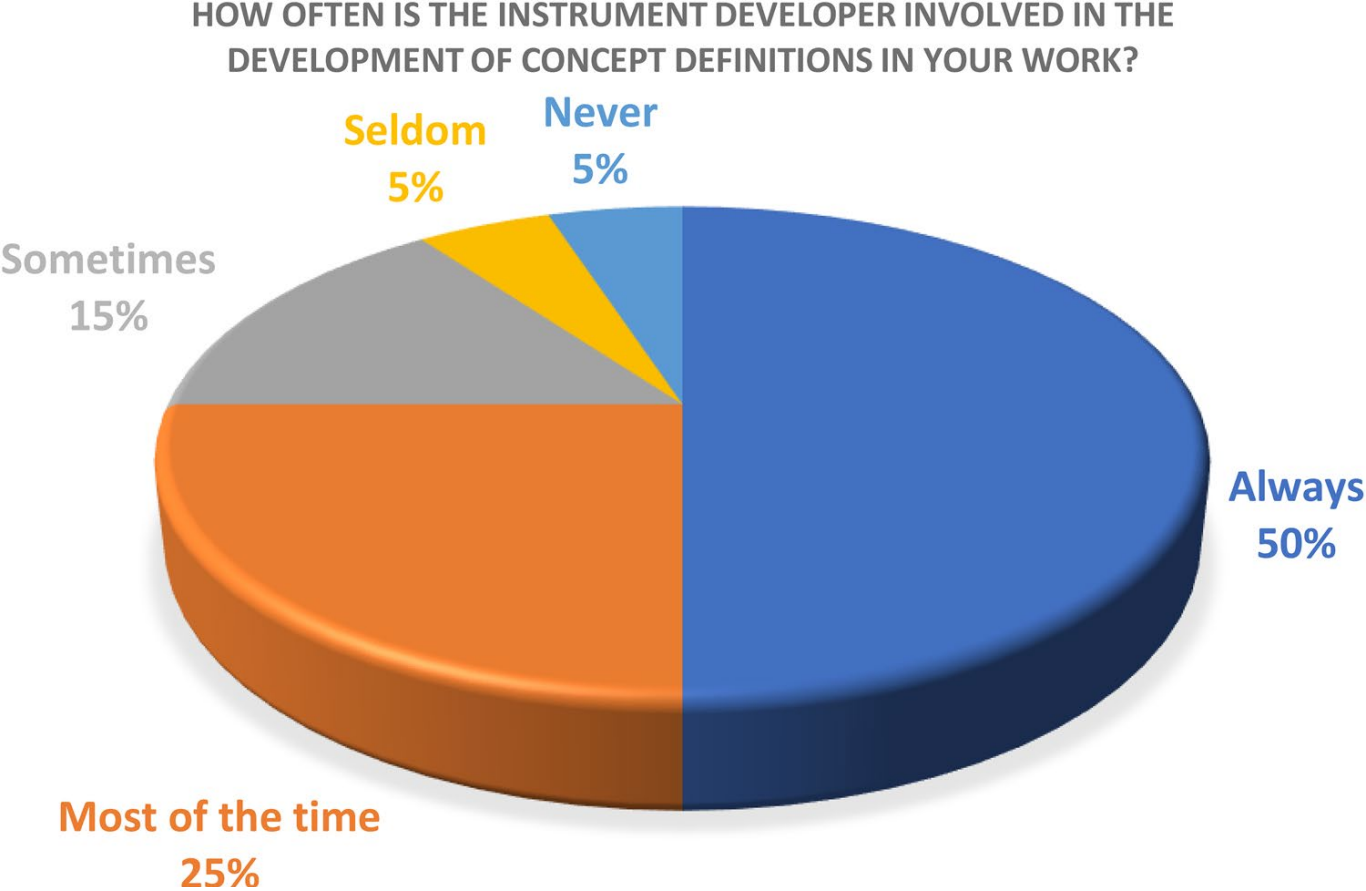
COA developer involvement

When asked how often the COA developer is involved in the creation of the concept definition document, the majority (15/20) of respondents reported having COA developer involvement either “Always,” or “Most of the time.” “Sometimes” was reported by 3/20 respondents. One respondent indicated “Seldom” having such involvement, and one respondent indicated “Never” having COA developer input in the creation of concept definitions (Fig. 5).

The majority (13/17) of respondents also indicated that there is no difference in the process followed or resources utilized if the developer of the COA is not available to contribute. One respondent commented that “if the developer cannot be involved then we at least ask our client to review and provide feedback.” Three respondents mentioned consulting a subject matter expert/expert reviewer in cases in which the COA developer cannot be involved, and two respondents mentioned review and approval by the client.

### Recommendations for the stakeholders involved in the process of defining concepts

Table 3 describes recommendations for the stakeholders involved in the process of defining concepts. Individuals with relevant training, experience, and expertise who have native-like proficiency in the source language should write concept definitions. This can range from the COA developer or development team to translation project managers. Other common roles and titles for people who might be involved in the generation of concept definitions include but are not limited to linguists, COA specialists, and survey research analysts, as it is important that concept definitions take into account not only linguistic insight, but also domain-specific information, i.e., the broader construct being measured such as fatigue or depression, and particularities about the how the COA is administered.

**Table 3:** Stakeholders involved in the process of defining concepts:

Stakeholder	Qualifications/Criteria	Rationale
COA Developer	Involved in the generation of the items in the COA for which definitions are being written	Able to provide information related to the intended meaning of each item in particular and to the fundamental conceptual framework of the COA
Reviewer (if COA developer not available or in addition to)	Subject matter expert – experience in disease condition Survey research expert – experience with COA research	Ability to provide insight into disease-specific information (e.g., symptoms) Ability to provide insight into measurement aspects such as equidistant response scales
Project Manager	Experience with linguistic validation and conceptual analysis of COAs Native-like proficiency in source language	Facilitates process and communication with COA developers and/or reviewers Correct interpretation (nuances, idioms, colloquialisms, etc.)

COA developer consultation and review of concept definitions are of critical importance as the developer has the most intimate knowledge of the concepts intended to be measured, information related to the conceptual framework of the COA, as well as to the history and intent of each item, instruction, recall period, and response option. If the COA developer cannot be involved, we recommend consulting an expert in the relevant subject matter. A subject matter expert would be an expert in the condition being evaluated and/or expert in survey research. Familiarity with linguistics, linguistic validation of COAs, and cross-cultural research issues are also helpful. Feedback from members of the target population (e.g., patients with the diagnosis targeted by the COA) is optional and can be included to the extent feasible.

### Results for aspects to include when defining concepts

**Fig. 6:**
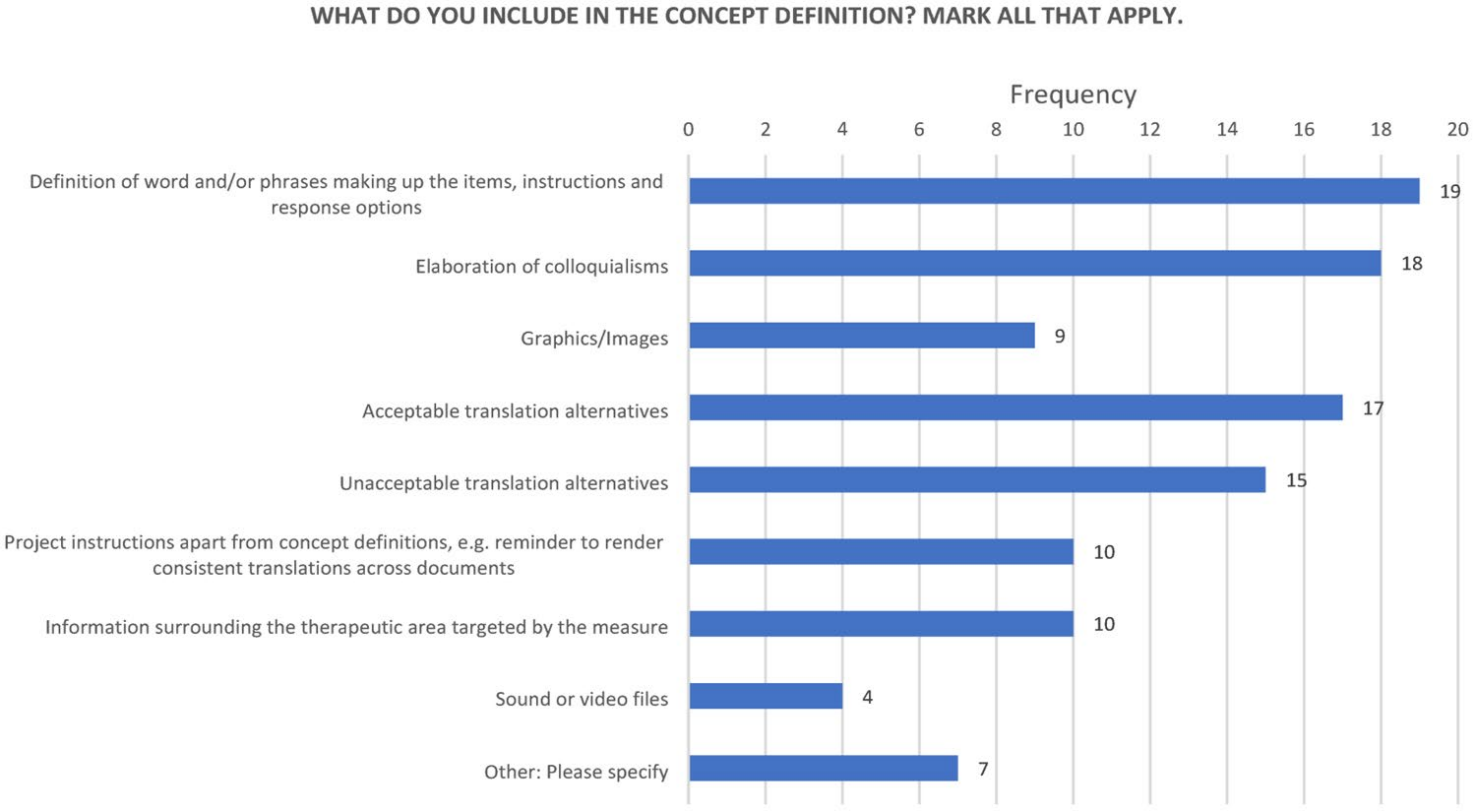
What to include in concept definitions

On the topic of what is included in a concept definition document, 19/19 respondents reported including the “Definition of word and/or phrases making up the items, instructions and response options.” Almost all (18/19) reported including “Elaboration of colloquialisms.” The great majority (17/19) reported including “Acceptable translation alternatives.” Slightly fewer (15/19) reported inclusion of “Unacceptable translation alternatives.” Slightly more than half (10/19) reported including “Information surrounding the therapeutic area targeted by the measure.” The same number (10/19) reported including “Project instructions apart from concept definitions,” for example, calls for translation consistency. Slightly less than half (9/19) reported including “Graphics/Images.” “Sound or video files” were endorsed by 4/19 respondents (Fig. 6).

**Fig. 7:**
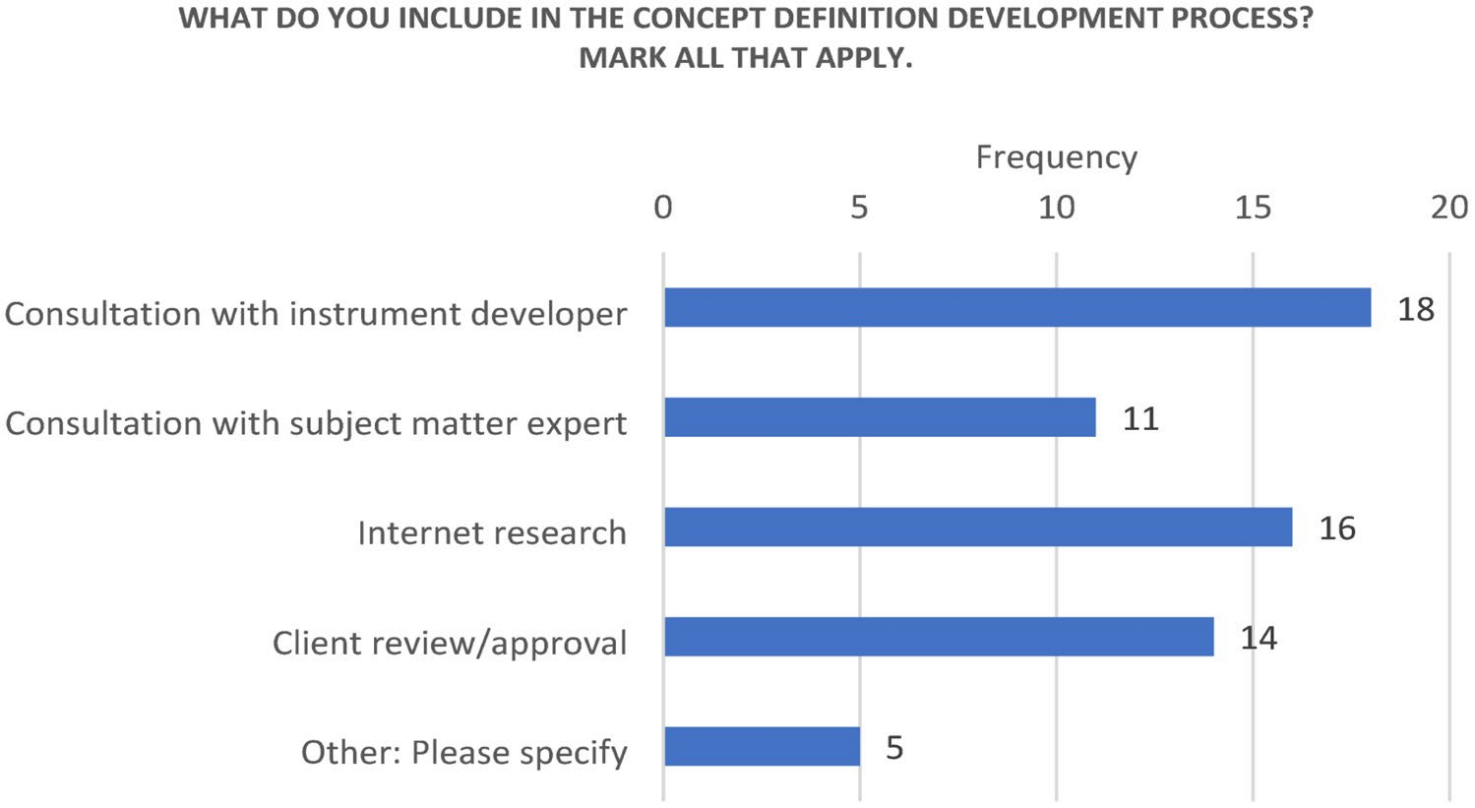
What to include in the concept definition process

When asked what is included in the concept definition development process, 18/19 participants reported “Consultation with instrument developer.” “Consultation with subject matter expert” was reported by 11/19. “Internet research” was reported by 16/19, and 14/19 respondents reported including “Client review/approval.” Inclusion of patients, clinicians and “logs from other language versions” was also mentioned in response to this question (Fig. 7).

### Recommendations for the aspects to include when defining concepts

Table 4 describes the aspects to include in a concept definitions document. The Working Group recommends that the content of the concept definitions include information related to the development, purpose, and context of the COA. This may include translatability assessment information and examples of acceptable translation alternatives or address other challenges with the content to be translated. Translation teams should ensure that concept definitions include information related to the therapeutic area targeted by the COA, as well as project-specific instructions such as calls for translation consistency and gender inclusivity, when appropriate. We recommend that concept definitions define phrases making up the items, instructions, recall period, and response options. Only concepts should be defined. Translation teams can define individual words when a standard dictionary definition is not helpful, is unrelated to the context of the COA, or when there is ambiguity about the intended meaning. Defining individual words is helpful when the same word is used multiple times with different meanings within the same COA.

**Table 4:** Aspects to include when defining concepts:

Aspect	Rationale
Information related to target therapeutic area	Important for translators to take into consideration
Information related to the COA development process	May help inform concept definition and reduce duplicate work in that concepts or rationale behind items may be described in the development process
Definition of concepts and domains	Ensures all parties agree on what is being measured
Elaboration of colloquialisms	Ensures all parties agree on what is being measured Colloquialisms and idioms may be particularly problematic to translate and harmonize across languages and regions
Acceptable/unacceptable translation alternatives	Encourages proactive discussion of text that might be difficult to translate and facilitates harmonization efforts across languages

Internet research can help inform definition of key terms and concepts as well as disease-/condition-specific context. Include explanations of colloquialisms, idioms, acceptable translation alternatives, and unacceptable translation alternatives to the extent they are known. Translation teams can include graphics/images, sound, or video files when available and helpful. Although there can be exceptions, the overall structure and order of the concept definitions should follow that of the source COA. The Working Group recommends updating concept definitions during the course of translation projects as new information becomes available and distributing the updated concept definition to the linguists translating the measure to ensure they use the most current version during the active translation phase.

Concept definition format may depend on developer preference, but the most widely used formats are MS Word, MS Excel, and other file formats that can be imported or exported from terminology management software (TMS) and/or databases. Word may be considered the easiest from a formatting and word processing point of view, while Excel is preferable for importing into databases, creation of searchable libraries/repositories, and for use with computer assisted translation (CAT) tools.

## Discussion

The Concept Definition Working Group of the ISOQOL TCA-SIG developed these recommendations for LSPs, translators, COA developers, project managers and sponsors/CROs, to address a lack of standardization in the process of concept definition development. In summary, translation teams should develop concept definitions during the preparation phase of the translation process if they do not exist. COA developers should be involved in the concept definition process whenever possible. This is key to ensuring information included in the concept definitions is as accurate and complete as possible. The Working Group recommends including a project manager with experience in linguistic validation and conceptual analysis of COAs. Include additional reviewers and/or subject matter experts if deemed necessary. Review by a subject matter expert is particularly important if the developer cannot be included in the process. Translation teams should include information related to the therapeutic area being studied, information related to the COA development process when available, definitions of concepts and domains, as well as elaboration of colloquialisms and acceptable/unacceptable translation alternatives in the concept definitions. The Working Group recommends centralizing the distribution of concept definitions to protect intellectual property and prevent duplication of efforts.

The Working Group considers the inclusion of patients in the process of concept definition as optional. This topic was raised in the context of the survey responses and manuscript review cycles and was discussed within the Working Group. It was decided that patient input should be reserved for the questionnaire development phase when concepts are generated by the development team. In the context of the translation and linguistic validation phase specifically, patient input should be reintroduced in the form of linguistic validation of the translated COA via cognitive interviews, a process in which the intended concept of each translated item, as defined by the concept definition, can be assessed and confirmed with the specific target population (e.g., patients diagnosed with the condition targeted by the COA who are native speakers of the target language).

One limitation of this study is the relatively small sample size made up of participants identified and recruited by the Working Group. Although the survey sample included a good mix of professionals representing LSPs, non-profit organizations, academia, COA developers and the pharmaceutical industry, there is always the possibility that other stakeholders may have different or unique opinions. Another limitation is the fact that the survey was carried out exclusively in English. Including non-English speaking stakeholders could have led to different results. In addition, no demographic information was collected to better describe the study sample.

Despite these limitations, the feedback from the survey sample combined with the expertise of the Working Group has resulted in a set of clear, actionable, and reasonable recommendations that have the potential to support the process of concept definition in the context of translation and linguistic validation of COAs. The results showed far more agreement and consensus than disagreement and discord, indicating a strong consensus across professionals in the area. The authors recognize that the field is changing faster than ever, and these changes could have an impact on the technologies used in the process of concept definition. Although the recommendations for concept definition will remain static, emerging technologies such as artificial intelligence could impact the process. For example, although the stakeholders involved and content required would not change, artificial intelligence, if properly trained, could potentially produce initial drafts of concept definitions to be reviewed and refined by humans in the loop.

Another potential limitation of the study is that the survey did not specifically ask whether there would be differences in the process according to COA type. The survey did not ask if the process would be different for a PRO than for a ClinRO, ObsRO or PerfO measure. Although additional research could be conducted to answer this question, the Working Group believes these recommendations would be adequate to cover all COA types. As an example, in the case of a ClinRO developed by a clinician, the developer would provide clinical insight, while other clinicians could be included as subject matter experts.

## Conclusions

Although good practices for the translation and linguistic validation of COAs have been in place for many years, the process for producing and maintaining concept definitions has not been adequately discussed nor standardized. Concept definitions are an important tool which can guide stakeholders in the process of ensuring COA translations are indeed meaningful and as equivalent as possible to the source language and across translated versions. Concept definitions inform possible alternative wording options, solutions to commonly encountered translation hurdles as well as what alternatives to avoid. The investment in time and resources necessary to create concept definitions is well worth the improved translation quality and project efficiencies gained later in the process, particularly in the context of harmonization across languages. Concept definitions are crucial to any successful translation process in that they ensure all parties involved in their development and usage (e.g., LSPs, translators, COA developers, project managers, sponsors/CROs) are aligned with the intended meaning of the items being translated. Without the conceptual equivalence that concept definitions promote, the patient voice cannot truly be heard.

The TCA-SIG Concept Definition Working Group set forth to standardize this important step in the translation and cultural adaptation process by listening to professionals with experience in the field. This led to the creation of these best practices which we deem to be a valuable contribution in the process of including the voice of the patient in health-related quality of life (HRQOL) research moving forward. The standardization of the development of concept definitions, with the goal of enhancing conceptual equivalence across translations, will ultimately support the pooling of data across countries in clinical trials and provide confidence that the data are comparable, interpretable and can be relied upon in evaluating clinical benefit of treatments.

## Appendix A

### Concept Definition Development in Translation Work

#### Project set up

1. How often is concept definition development required for the translation and linguistic validation of clinical outcome assessment (COA) measures in your work?

a. Always

b. Most of the time

c. Sometimes

d. Seldom

e. Never

2. Do you refer to the concept definitions document by another term, e.g., concept elaborations?

a. No

b. Yes: Please specify ________________________

3. How many individuals are involved in the concept definition development for 1 COA?

a. 1

b. 2

c. 3

d. 4 or more

4. Who is involved in the development of concept definitions in your work? Mark all that apply:

a. Project manager

b. In-house linguist

c. Outside vendor specialist/linguist

d. Instrument developer

e. Other: Please specify ________________________

5. How often is the instrument developer involved in the development of concept definitions in your work?

a. Always

b. Most of the time

c. Sometimes

d. Seldom

e. Never

6. How often is the concept definitions developer a native speaker of the source language in your work?

a. Always

b. Most of the time

c. Sometimes

d. Seldom

e. Never

f. Not sure

7. How often is the source language of the clinical outcome assessment a language other than English in your work?

a. Always

b. Most of the time

c. Sometimes

d. Seldom

e. Never

f. Not sure

8. Do you investigate whether a concept definition document is available prior to developing a new one?

a. No

b. Yes: Please specify ________________________

9. Do you maintain a repository of concept definitions?

a. Yes

b. No

#### Concept definitions development process

10. What format is used for concept definition document in your work?

a. Word table

b. Excel spreadsheet

c. Other: Please specify ________________________

11. How is the concept definition document structured?

a. In the exact order of the source measure

b. By category, e.g., colloquialisms, instructions, response options

c. Other: Please specify ________________________

12. What do you include in the concept definition document? Mark all that apply:

a. Definition of word and/or phrases making up the items, instructions and response options

b. Elaboration of colloquialisms

c. Graphics/Images

d. Acceptable translation alternatives

e. Unacceptable translation alternatives

f. Project instructions apart from concept definitions, e.g., reminder to render consistent translations across documents

g. Information surrounding the therapeutic area targeted by the measure

h. Sound or video files

i. Other: Please specify ________________________

13. What do you include in the concept definition development process? Mark all that apply:

a. Consultation with instrument developer

b. Consultation with subject matter expert

c. Internet research

d. Client review/approval

e. Other: Please specify ________________________

14. Are there any differences in the process you follow, or the resources you utilize when the instrument developer is not involved in concept definition development?

a. Yes

b. No

 Please explain your answer in a sentence or two:

15. How are updates made to the concept definition document once the translation is underway?

a. As needed with document version control.

b. Post-project.

16. If there are any other comments you would like to contribute relative to the concept definition development process, please write those comments here.

Thank you for your participation. Please provide the name of the company or organization you represent below.

## Electronic supplementary material

Below is the link to the electronic supplementary material.

Supplementary Material 1

## Data Availability

By request to the corresponding author.

## Abbreviations

ClinRO: Clinician-Reported Outcome
COA: Clinical Outcome Assessment
CRO: Clinical Research Organization
eCOA: electronic Clinical Outcome Assessment
EMA: European Medicines Agency
FDA: The U.S. Food and Drug Administration
HRQOL: Health-Related Quality of Life
ICMJE: International Committee of Medical Journal Editors
ISOQOL: International Society for Quality of Life Research
ISPOR: International Society for Pharmacoeconomics and Outcomes Research
LSP: Language Service Provider
ObsRO: Observer-Reported Outcome
PerfO: Performance Outcome
PRO: Patient-Reported Outcome
PROM: Patient-Reported Outcome Measure
TCA-SIG: ISOQOL Translation and Cultural Adaptation Special Interest Group
